# Oral food challenges in a specialised allergy outpatient clinic in Sao Paolo, Brazil

**DOI:** 10.1186/2045-7022-5-S3-P78

**Published:** 2015-03-30

**Authors:** Rozalem Ana Carolina, Renata Cocco Rodrigues, Lucila Oliveira Camargo Lopes, Marcia Mallozi Carvalho, Dirceu Sole

**Affiliations:** 1 Federal University of Sao Paolo, Sao Paolo, Brazil

## Background

Oral food challenge (OFC) is the most reliable method to diagnose food allergy in suspected children. The aim of this study was to describe the profile of patients undergone to OFC in the Division of Allergy and Clinical Immunology, Federal University of Sao Paulo, especially for cow/s milk (CM).

## Methods

A retrospective study of chart analysis of 171 patients undergone to 220 OFC, between June/2007 and Feb/2014. Food tests comprised CM, egg, soy,peanuts, nuts, seafood, meat, chicken, tartrazine, chocolate, wheat and coconut. Patients were evaluated according to the type of CM's challenge (open or double-blind placebo-controlled), aim of the procedure (diagnosis or follow up tolerance), symptoms, body mass index, time of breastfeeding, age at first reaction, family history of food allergy, presence of other atopic diseases (asthma, allergic rhinitis and atopic dermatitis) and skin prick test.

## Results

65% of patients were male (n=111) with median age of 3 years and 2 months. The most common tested foods were CM (n=148), egg (n=22) and soy (n=19). CM's challenges were: negative in 109 (74%), inconclusive in 4 (3%) and positive in 34 (23%) tests (27 open food challenge and 7 double-blind placebo-controlled). From patients who had a positive OFC, 52% had referred cutaneous symptoms, 22% gastrointestinal symptoms, 19% respiratory symptoms and 7% claimed to have had anaphylaxis. When they undergone to OFC, cutaneous symptoms were observed in 68% and 5% had an anaphylaxis episode. 35% of children elicited symptoms after less than 1ml ingestion of CM. 67% of all reactions were classified as mild (skin and/or upper respiratory tract) and 18% as severe reaction (skin and lower respiratory tract). Among the group that passed OFC, 17% (n=18/109) were in a CM free diet just because of wheezing or recurrent respiratory tract infection.

## Conclusion

Although there are some difficulties in performing OFC in the routine, there are multiple discrepancies between referred symptoms and diagnosis of food allergies. CM responds for the great majority of food allergy in this population.

